# Embolisation of a high – flow renal arteriovenous fistula with the use of simultaneous transvenous and transarterial approach and balloon-assisted coil embolization

**DOI:** 10.1186/s42155-024-00451-9

**Published:** 2024-04-19

**Authors:** D. Markoutsas, D. Tzavoulis, G. Tsoukalos, I. Ioannidis

**Affiliations:** 1 Department of Interventional Radiology, Athens Naval Hospital, 70 Dinokratous Str., Athens, 11521 Greece; 2 Department of Interventional Radiology, Hygeia Hospital, 4 Erithrou Stavrou Str., Marousi, Athens, 15223 Greece; 3 Radiology Department, Larissa University Hospital, Mezourlo Area, Larissa, 41110 Greece

**Keywords:** Renal arteriovenous fistula, DSA, Embolisation

## Abstract

**Background:**

Renal arteriovenous fistula (RAVF) is a rare vascular malformation, which can be asymptomatic or may cause hemorrhage, hypokalaemic hypertension, heart failure and hematuria. Endovascular embolization is a minimally invasive method which can preserve renal parenchyma. In our case, balloon assisted coil embolization with simultaneous transvenous and transarterial approach was used. A remodelling balloon, which is routinely used in neurovascular procedures, was chosen in order to eliminate the risk of coil migration and preserve feeding artery and renal parenchyma.

**Case presentation:**

We present a case of successful balloon – assisted coil embolization of a high flow renal arteriovenous fistula in a 25-year-old male patient via simultaneous transarterial and transvenous approach with preservation of the feeding artery.

**Conclusion:**

Endovascular embolisation is a safe and effective treatment of RAVFs with low risk of complications. Simultaneous transarterial and transvenous coil deployment with the use of a flow control balloon catheter can eliminate the risk of coil migration and coil protrusion into the parent artery with permanent RAVF occlusion and renal parenchyma preservation.

## Introduction

Renal arteriovenous fistula (RAVF) is a rare vascular malformation in which there is a direct communication between the feeding artery and the draining vein, without the interference of capillary vessels.

RAVF can be classified into traumatic and non-traumatic shunts. Traumatic RAVFs are mainly caused by iatrogenic injury, especially percutaneous biopsy. Non-traumatic RAVFs are generally classified as congenital, acquired or idiopathic [1, 2]. Idiopathic RAVF is the rarest type [3].

RAVFs may be clinically asymptomatic or can be associated with several symptoms such as retroperitoneal hemorrhage, high—output congestive heart failure, hematuria, pain and high pressure due steal effect from renal parenchyma and renin – aldosterone system activation [1, 4].

Computed tomography, color doppler ultrasound and digital subtraction angiography (DSA) are valuable diagnostic tools for the detection of RAVFs. Renal arteries DSA is regarded as the gold standard diagnostic tool because it can determine the fistula location, renal blood supply and hemodynamic changes [5].

Angiographically RAVFs can be classified into three types according to a classification proposed by Cho et al. Type I includes RAVFs consisting from a single or a few arteries (fewer than four) shunted into a single draining vein. Type II includes RAVFs consisting from multiple arterioles shunted into a single draining vein and type III includes RAVFs consisting from multiple shunts from multiple arterioles and venules formatting a complex vascular network. Furthermore, type III RAVFs can be subdivided into two categories according to the size of vascular channels; IIIa (nondilated), IIIb (dilated) [6].

Therapeutic armamentarium has been evolved from partial or total nephrectomy to endovascular embolisation. Indications for treatment include hypertension, cardiac failure, recurrent or persistent hematuria, hemodynamic effects associated with the RAVF, progressive increase in fistula size. Endovascular embolisation has the ability to preserve renal parenchyma and renal function with fewer complications and therefore is now the preferred option. Surgical approach is considered when endovascular intervention seems unfavorable [1, 7].

We report a case of a 25-year-old male diagnosed with a high flow right RAVF who underwent endovascular embolisation with a simultaneous arterial and venous approach.

## Case presentation

**Fig. 1:**
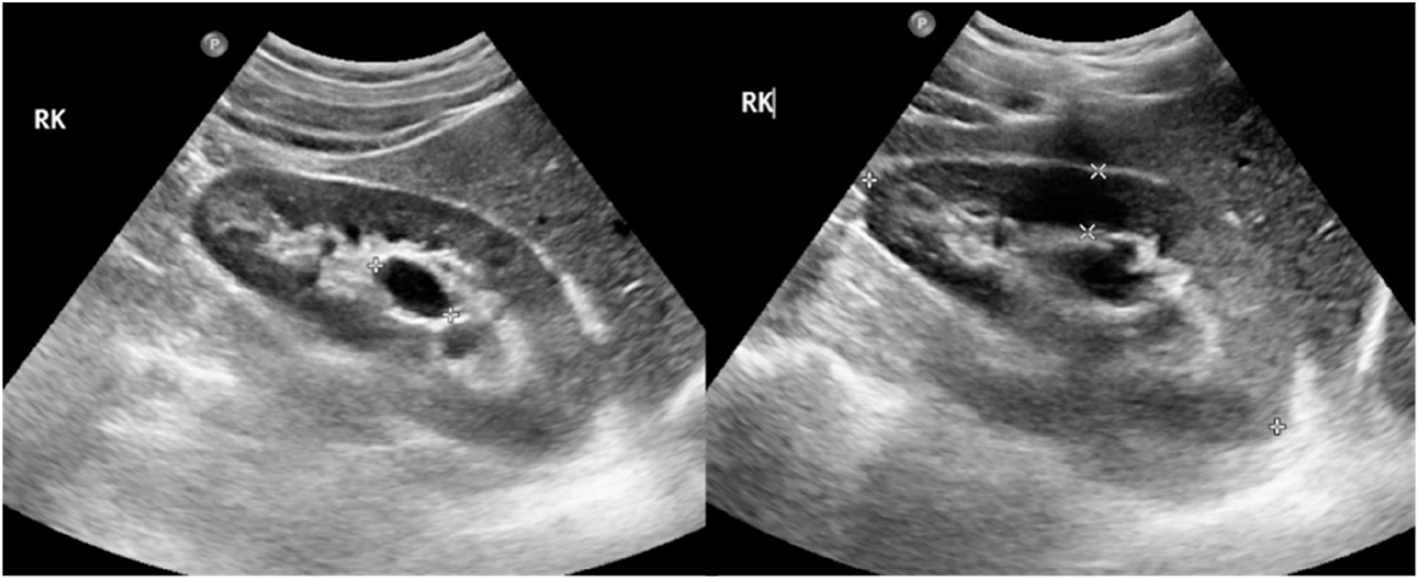
Ultrasound images of the right kidney showing cystic—like lesions

A 25-year-old male presented to our department with two episodes of gross hematuria. Personal and family medical history were negative of any underlying diseases. Ultrasound revealed lobulated paracalyceal cystic – like lesions in the parenchyma of the right kidney with no dilation of its pelvicalyceal system (Fig. 1).

**Fig. 2:**
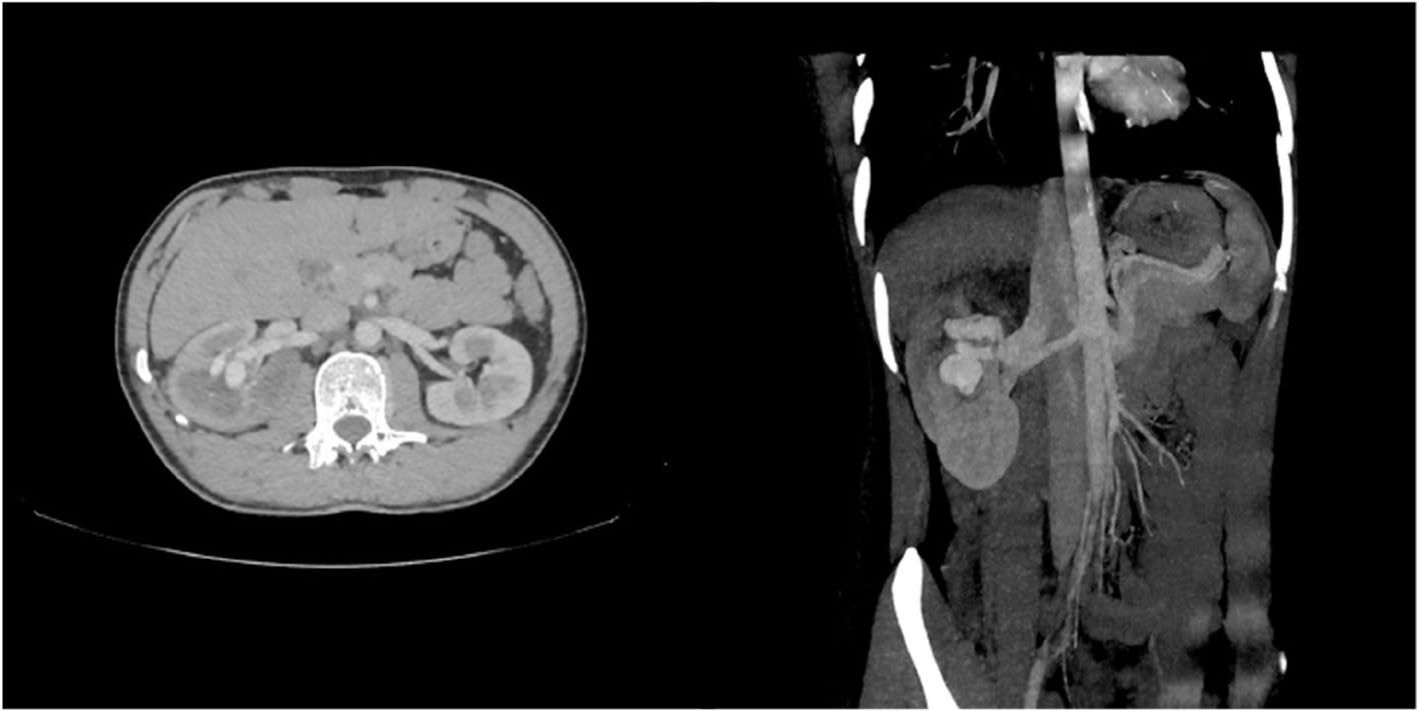
Contrast enhanced computed tomography axial and coronal plane with MIP reconstruction demonstrating a right RAVF

Contrast enhanced computed tomography in the arterial phase demonstrated dilated upper lobe interlobar artery of the right kidney and early enhancement of a variceal venous structure which drained in the right renal vein, findings indicative of renal arteriovenous fistula (Fig. 2). There was no history of trauma or medical procedure, so the RAVF was considered as non -traumatic. Fistulous site was measured approximately 12 mm.

**Fig. 3:**
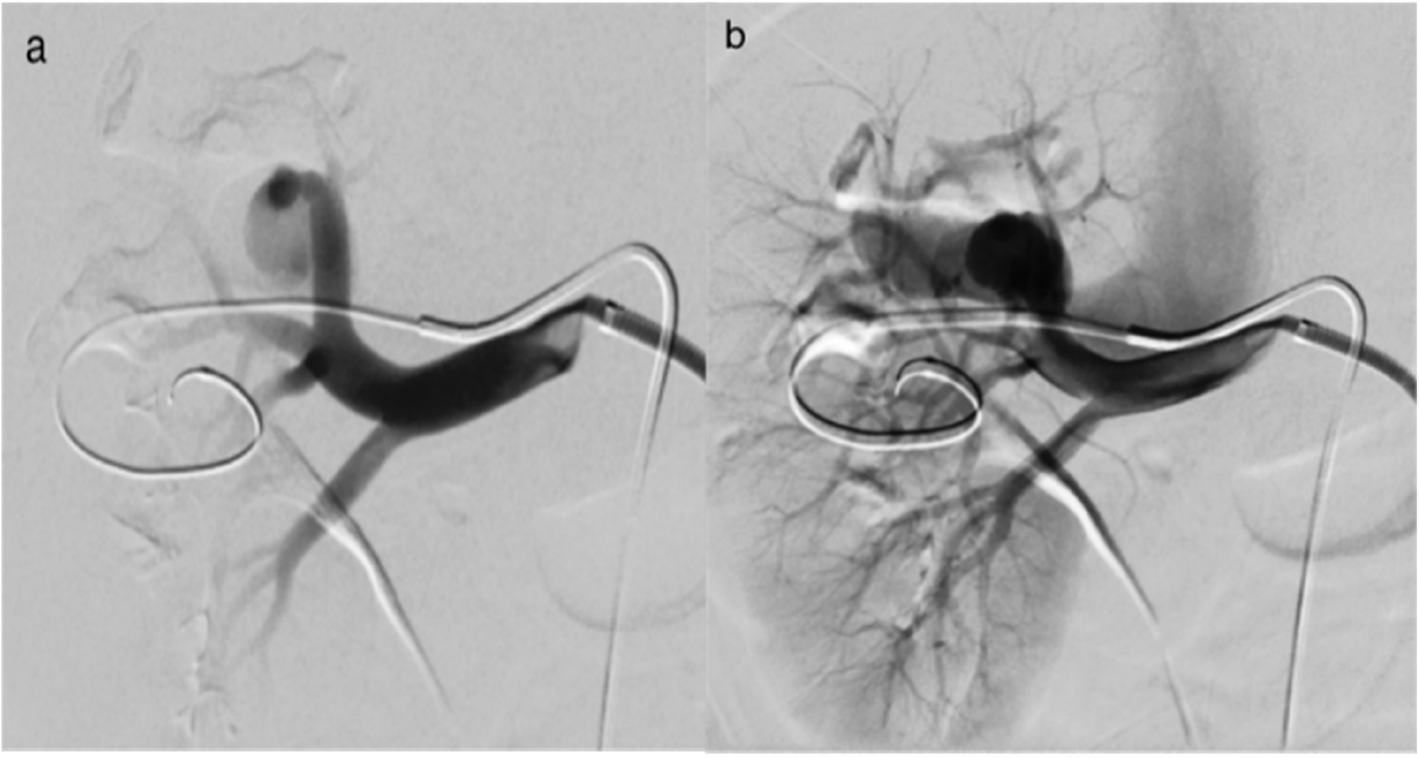
**a**, **b** Right main renal artery angiography reveals an enlarged upper pole interlobar artery, the direct communication, and early opacification of a dilated vein and IVC, findings indicative of a RAVF

The procedure was conducted in the cath lab (Siemens Artis Zee) under aseptic conditions and local anesthesia. Patient was in supine position and vital signs were monitored throughout the procedure. Under ultrasound guidance puncture of the right common femoral artery was performed and a 5F vascular sheath was introduced. A pre – shaped 5F Cobra catheter (Merit Medical) was advanced. Then, a 6 F × 45 cm Ansel guiding sheath (Cook Medical) was advanced into the proximal right renal artery. DSA revealed an enlarged interlobar renal artery and early opacification of an enlarged draining vein and inferior vena cava (IVC), findings indicative of type I RAVF (Fig. 3).

Next, under ultrasound guidance, puncture of the right common femoral vein was performed and a 5F vascular sheath was introduced. A pre—shaped 5F Simmons catheter (Merit Medical) was advanced into the right renal vein and selective catheterization of the dilated vein was performed with the help of a hydrophilic guidewire (Terumo). Super – selective catheterization of the venous pouch adjacent to the feeding artery was achieved by a 2,7F microcatheter (Progreat, Terumo).

**Fig. 4:**
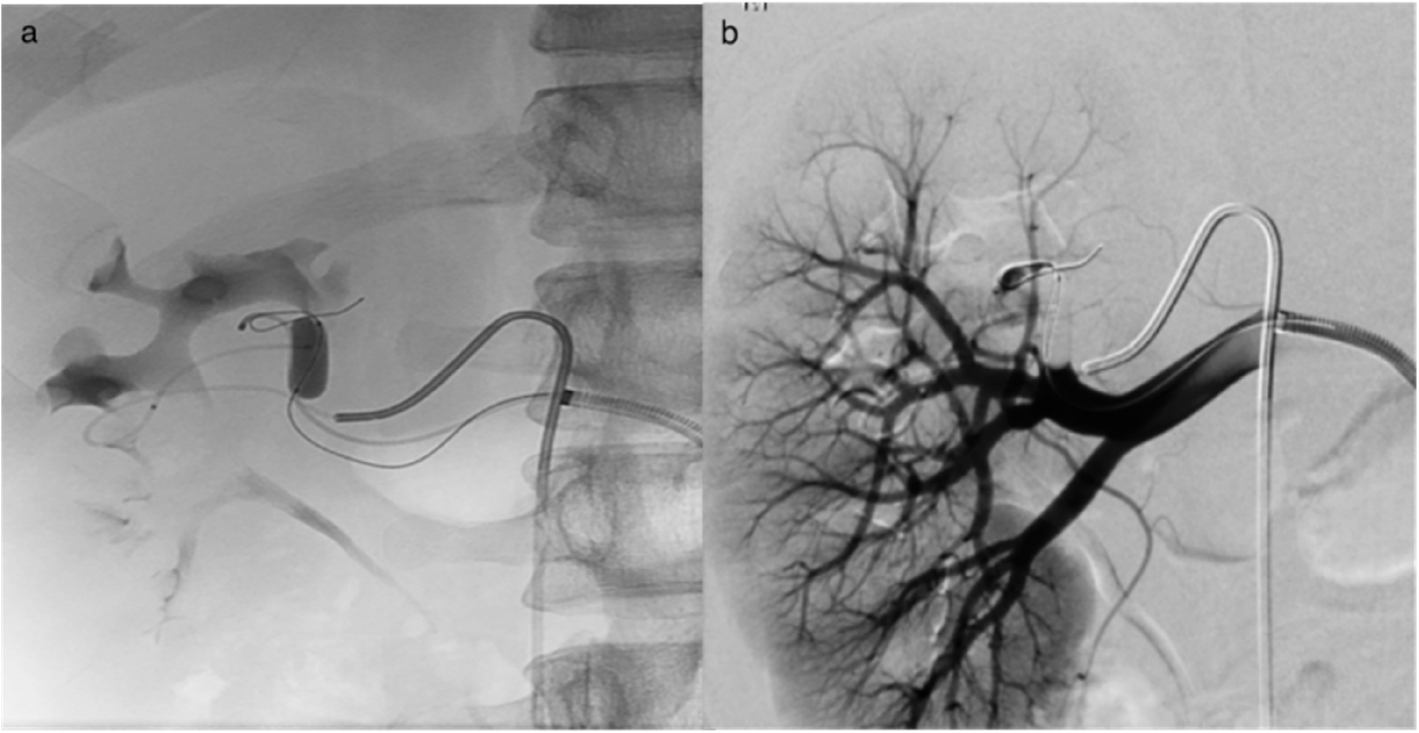
Fluoroscopic image (**a**) with the balloon inflated shows dual microcatheter approach and balloon catheter into the feeding artery. Right main renal artery angiography (**b**) with the balloon inflated within the feeding artery shows the accurate site of AVF

**Fig. 5:**
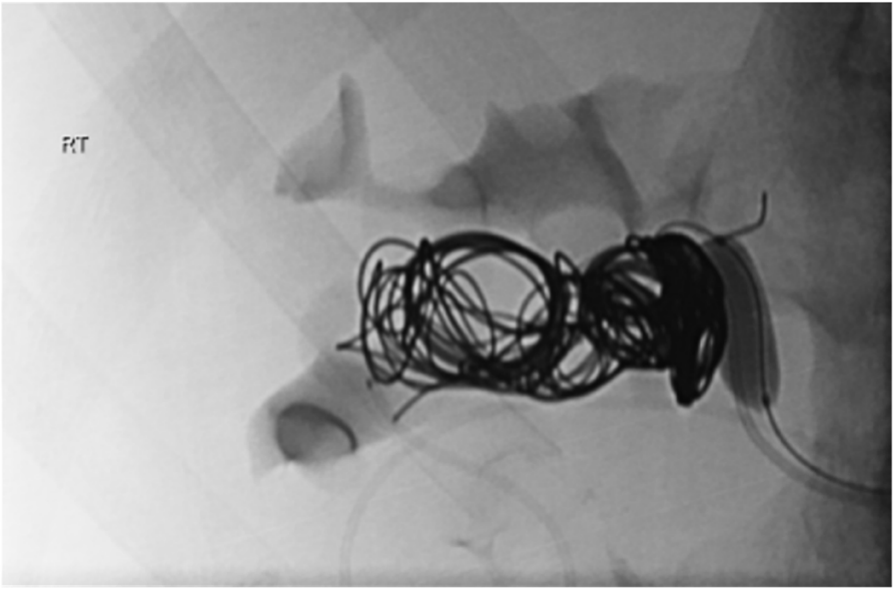
Simultaneous transarterial and transvenous coil deployment

Then, a Hyperform 7 × 15 mm balloon (Medtronic) was advanced into the feeding artery across the site of the fistula. Selective angiogram with the balloon inflated showed no opacification of the fistula and the accurate site of the communication was documented and type I RAVF was confirmed. A 2,6 F microcatheter (Asahi Intecc) was advanced at the venous pouch at the site of the fistula (Fig. 4) and multiple electrically detachable metallic coils (Optima/OptiMAX/BALT) were simultaneously deployed through the transarterial and transvenous microcatheter into the fistula initially with the balloon inflated (Fig. 5). In particular, there were used three coils 18 × 20 mm, one 14 × 47 mm, one 13 × 43 mm, one 12 × 40 mm, two 10 × 30 mm, one 10 × 17 mm and one 10 × 13 mm. Balloon inflation prevents coil migration and protrusion of the coils into the feeding artery.

**Fig. 6:**
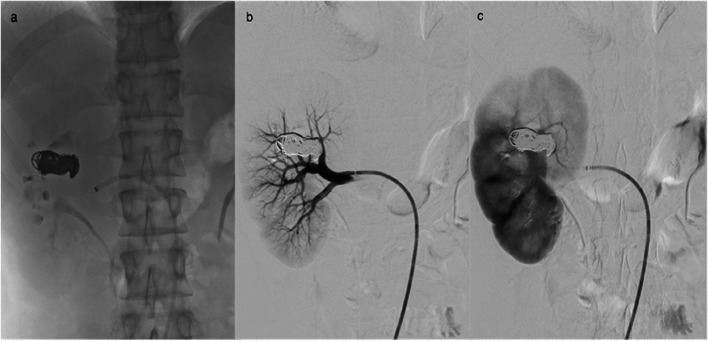
Fluoroscopic image (**a**) showing deployment of detachable coils into the fistula. DSA arterial (**b**) and nephrographic (**c**) phase showing total occlusion of the RAVF with feeding artery and renal parenchyma preservation

The final result was total occlusion of the fistula with preservation of feeding artery and the renal parenchyma (Fig. 6).

There were no minor or major complications. Patient was discharged the following day in good condition, with the instruction of a new contrast enhanced computed tomography after 6 months.

## Discussion

RAVFs represent an abnormal communication between the renal arterial and venous system, usually located in the collecting system and not in the renal parenchyma, with most of the case in the renal upper pole. The first RAVF was reported by Vorela in 1928 [8]. Also, RAVFs are more often observed in 30 -40 year-old women, mostly in the right kidney than the left [8, 9].

The aim of a successful endovascular treatment of a RAVF complete and permanent occlusion of the fistula while preserving as much normal renal parenchyma as possible [6]. Embolic materials used for the treatment of RAVF are gelfoam (gelatin sponge particles), absolute and polyvinyl alcohol, coils, vascular plugs, n-buttyl 2 – cyanoacrylate (NBCA) glue and ethylene vinyl alcohol copolymer (Onyx) [6, 10].

In our case we had to deal with a high flow type I RAVF which increases the risk of coil migration. To achieve a stable coil position, we decided to deploy detachable coils simultaneously via transarterial and transvenous route to allow accomplishment of high coil volume within the fistula as quickly as possible. Hyperform is a single lumen compliant balloon which is mainly used for brain aneurysm embolization with balloon remodeling technique [11]. The balloon was used to control the blood flow and protect the feeding artery during the placement of coils. The use of Hyperform remodeling balloon catheter was used to prevent the coil protrusion in the feeding artery as well coil migration.

In a case by Fung et al. simultaneous arterial and venous approach was used with coil deployment and use of NBCA. In that case dual approach was used because of coil migration into the IVC during transarterial coil deployment [4]. NBCA is an adhesive liquid which polymerizes immediately on contact with anions in the blood, thus occluding the affected vessel. It is mixed with lipiodol to control polymerization time and it is mostly used in type III RAVF. Onyx can be an alternative option, which is less thrombogenic than NBCA and required more prolonged period of injection [6]. In this particular case, total occlusion of the fistula was achieved only by the coil’s deployment. Furthermore, we decided not to use a liquid embolic material in order to prevent the risk of migration.

Vascular plugs can be valuable tools for treating type I RAVFs and they are associated with low risk of migration. The disadvantage of vascular plugs is that they require larger catheter tip and stiff delivery wire system which can be difficult to advance through tortuous access route. In a case by Castellano et al., a RAVF was successfully occluded with the use of a 4 -mm vascular plug [12]. We preferred the dual microcatheter technique because of the anatomy of the RAVF. Also, vascular plugs occlude the feeding artery of the AVF. With the use of the remodeling balloon, we managed to preserve the feeding artery which was our purpose from the beginning of the procedure.

Finally, in a case described by Chen et al., an atrial septal defect occluder was used in congestion with coils an vascular plug to occlude a large RAVF with promising results [3].

## Conclusion

Endovascular embolisation is a safe and effective treatment of RAVFs with low risk of complications. Simultaneous transarterial and transvenous coil deployment with the use of a flow control balloon catheter can eliminate the risk of coil migration and coil protrusion into the parent artery with permanent RAVF occlusion and renal parenchyma preservation.

## Data Availability

Not applicable.
